# Optical deep-cortex exploration in behaving rhesus macaques

**DOI:** 10.1038/s41467-021-24988-8

**Published:** 2021-08-02

**Authors:** Masanori Matsuzaki, Teppei Ebina

**Affiliations:** 1 Department of Physiology, Graduate School of Medicine, The University of Tokyo, Tokyo, Japan; 2 Brain Functional Dynamics Collaboration Laboratory, RIKEN Center for Brain Science, Saitama, Japan; 3 International Research Center for Neurointelligence (WPI-IRCN), The University of Tokyo Institutes for Advanced Study, Tokyo, Japan

**Keywords:** Brain-machine interface, Motor cortex

## Abstract

Two papers published in June 2021 used a two-photon microscope or one-photon miniature microscope to interrogate the motor cortex in behaving macaque monkeys. The imaging was performed over several months, and the direction of natural arm reaching was decoded from the population activity.

Non-human primates (NHPs) are important animal models for understanding higher brain functions relevant to complex human behaviors including cognition and motor control. In particular, rhesus macaques have been used for electrophysiological and anatomical studies. However, when electrodes, even multi-electrode arrays, are used for neuronal recording, it is difficult to simultaneously measure the activity of hundreds of neurons in local circuits. Furthermore, it is currently not technically possible to identify the location and morphology of multiple recorded neurons, identify the neuronal subtype, and confirm that the exact same neuron is recorded over a period of weeks to months. By contrast, calcium imaging via fluorescence microscopy is suitable for gathering longitudinal data on population activity in local circuits with the identification of single neurons. Calcium imaging is widely used in rodents, fish, and small invertebrates but its applications in behaving NHPs are limited^1^. This is because expression of genetically encoded calcium indicators (GECIs) is generally low (the reason for this is not clear) in the primate cortex and light scattering prevents imaging in deeper brain regions. Furthermore, chronic implantation of optical devices (cranial window or lens) in the large brain with minimal cortical damage is challenging and motion artifacts induced by arm movement, pulsation, and respiration need to be strictly suppressed. Despite these difficulties, two-photon imaging of GCaMP-expressing neurons in the motor cortex in head-fixed condition and one-photon calcium imaging of the motor cortex with a miniature microscope (miniscope) in a non-head-fixed condition during arm-movement tasks were established^2,3^ in a small NHP, the common marmoset. Two recently published papers overcame these challenges now also in the behaving macaque with a larger brain.

Trautmann et al.^4^ used two-photon microscopy to image dendritic calcium signals in the rhesus macaque motor cortex. Two-photon microscopy has a high spatial resolution allowing it to resolve not only single neurons, but also single dendrites and axons. The authors took advantage of this to image the activity of individual apical dendrites (or axons) in layer 1 (L1) that extended from neurons in superficial and deep layers. During imaging, a glass window attached to a tissue stabilizer was placed on an artificial dura inside an implantable chamber to restrict tissue motion via gentle pressure on the cortical surface (Fig. 1a). This tissue stabilizer was fixed to a head stabilizer. This head-fixation system allowed detecting fluorescence changes in small dendritic/axonal structures in arm-reaching monkeys with minimal motion artifacts. The cranial window was placed on the dorsal premotor cortex (PMd) and primary motor cortex (M1), and an area of ~12 mm in diameter was accessible for imaging. The field of view (FOV) was ~700 × 700 μm and the frame rate 30 Hz. GCaMP expression was achieved by injection of adeno-associated viruses (AAVs).

**Fig. 1:**
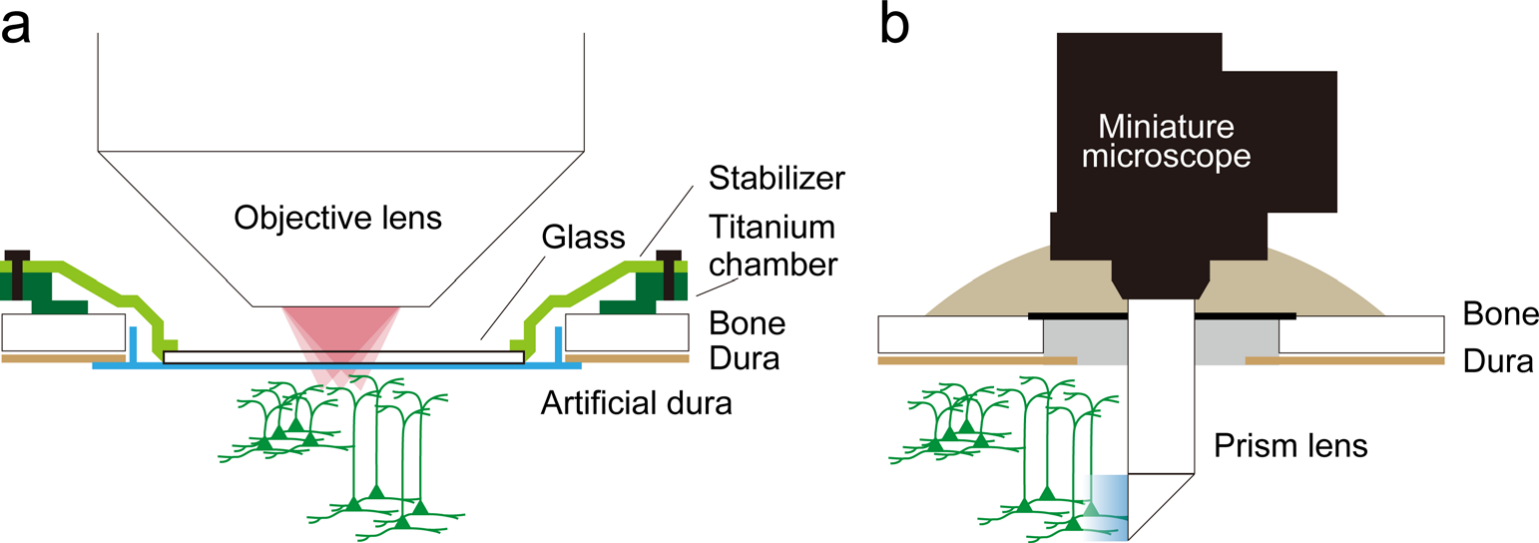
Two configurations of calcium imaging in the macaque motor cortex. **a** Configuration of two-photon imaging by Trautmann et al. It is possible to image the activity of multiple dendrites in the superficial layer of the cortical region under a large cranial window. The same and/or different fields can be imaged across days. The head of the macaque should be fixed. **b** Configuration of one-photon miniscopic imaging by Bollimunta et al. Chronic calcium imaging of multiple neurons in the same deep-layer field is possible. This is applicable to free-moving macaques.

Imaging was conducted while the monkey reached with its arm for one of four targets. The authors found ~120 regions of interest (ROIs) that putatively corresponded to dendritic or axonal processes in each FOV of M1. Approximately 50% of these ROIs showed direction-selective responses during arm movement, with their selectivity being stable within each session. The same field with similar neural structures was repetitively imaged using the vasculature structure for reference. The authors constructed an online decoder to predict the reach direction from the raw pixel values; the online decoder performance was high: ~87% with two targets and ~70% with four targets. Furthermore, the authors cleared the imaged tissue volume using CLARITY^5^ and demonstrated that the imaged dendrites could be traced to the neuronal somata in layer 5 at a depth of 1.5 mm from the cortical surface.

Bollimunta et al.^6^ used a one-photon miniscope^7^ for calcium imaging in the PMd of behaving rhesus macaques. A prism lens of 1.0 mm diameter was implanted in the PMd with the distal end being 2 mm below the cortical surface bilaterally, and a baseplate attached to the lens was sturdily fixed on the skull with cement (Fig. 1b). Immediately before imaging, the main body of the microscope could be easily docked to the baseplate. The FOV was 750 × 900 μm and the frame rate was 10 or 20 Hz. Thanks to the prism, the FOV was perpendicular to the cortical surface and the superficial region above the imaged region was undamaged. The device was stably secured and did not induce inflammatory activity. The same FOV was stably imaged over at least eight months, with ~100 active neurons being imaged in each session. Using a cell registration algorithm, many neurons could be tracked across sessions, and 17 active neurons were tracked across seven imaging sessions over three weeks.

The left PMd was imaged while the head-unfixed monkey sat in a chair and reached for either a left or right target with the right arm. Approximately 75% of neurons showed reach-related activity, and ~30% showed a direction-selective response. The direction selectivity in each neuron was stable over more than two weeks. Furthermore, the authors took advantage of the small size of the miniscope to simultaneously dock two miniscopes to the baseplates in the left and right hemispheres and image the PMd activity. They found that many neurons exhibited contralateral reach-related activity, while a minority of neurons were sensitive to ipsilateral reaches. The authors also showed that AAVs carrying a tetracycline-inducible gene amplification system are effective in the macaque motor cortex, as they are in the macaque visual cortex and marmoset sensorimotor cortex^2,3,8,9^.

These studies mark the first important steps in observing the population activity in local circuits in behaving macaques. The points in common are that populations of neurons can be simultaneously imaged and the same fields can be tracked over weeks to months. The imaging conditions (FOV size, frame rate, and number of ROIs) are also comparable between both studies. The stable direction selectivity across sessions suggests that the imaged regions were healthy and motion artifacts were negligible. Thus, both techniques can be applied to many already-established cognitive and motor control tasks in the macaque. In the mouse, when calcium transients in individual M1 neurons are used to deliver a reward in real-time, mice can change the activity of target neurons^10,11^. Such experiments may be applied to advance brain-computer interfaces in the macaque.

One of the next technical steps is to label specific types of neurons. Even when cell-type-specific promoters in the macaque cannot be used, projection area-specific neurons could be retrogradely labeled. It is also possible to image long-range axons of neurons in the AAV-injected region. For example, imaging of these neurons may reveal which types of neurons show the plasticity required for brain-computer interfaces. Multi-color GECI imaging shows promise for the simultaneous imaging of different types of neurons. If green GECI-labeled long-range axons and red GECI-labeled neurons in the axonal projecting areas are imaged with the miniscope, the inter-areal interaction dynamics could be examined. Using two-photon imaging, the long-range axons and their postsynaptic dendrites in L1 may be simultaneously imaged at single-neuron resolution. The combination of these imaging methods with cell-type-specific labeling would also enable to perform precise optogenetic stimulation to provide important insights into the functions of these neurons in behaviors.

Another next step is to expand the imaging field. A large cranial window of more than 10 mm has now been set on the macaque brain for two-photon imaging. Wide-field one-photon imaging through such a cranial window may be used to detect population-averaged activity^12^. Then, the transformation from motor preparatory activity to motor execution activity in individual PMd and M1 neurons could be imaged using two-photon imaging. In addition, the use of contiguous FOVs for two-photon imaging is expanding^13^. Although the imaging area of the miniscope is fixed, multiple miniscopes can be set, as Bollimunta et al. demonstrated. The miniscope, but not two-photon microscopy, may be used to image neurons buried in the cortical sulci, such as those in the intraparietal cortex. Thus, trial-by-trial dynamics in population activity between the motor cortex and parietal cortex during motor adaptation could be clarified. A miniscope with a long lens should be suitable for imaging subcortical regions such as the striatum, thalamus, and ventral tegmental area. Other genetically encoded probes able to sense the release of monoamines such as dopamine may also be imaged^14^. Two-photon imaging and miniscopic imaging are not antagonistic but complementary in macaque research, as in rodent research. Through sharing the latest GECIs, other probes, and image processing algorithms, they can be used hand in hand to develop their respective strengths.

The above-mentioned imaging technologies will help us understand the changes in cortical population dynamics before symptoms appear and during the chronic stages of disorders, as well as during recovery after treatment starts. Furthermore, because the development of transgenic macaque and marmoset models for neurodegenerative and psychiatric disorders is progressing, subject to bioethical considerations^15^, fluorescence imaging technologies in NHPs will help to advance basic and clinical neuroscience research.
